# Strain-level profiling with picodroplet microfluidic cultivation reveals host-specific adaption of honeybee gut symbionts

**DOI:** 10.1186/s40168-022-01333-9

**Published:** 2022-08-31

**Authors:** Yujie Meng, Shuang Li, Chong Zhang, Hao Zheng

**Affiliations:** 1 College of Food Science and Nutritional Engineering, China Agricultural University, Beijing, 100083 China; 2 Department of Chemical Engineering, Institute of Biochemical Engineering, Tsinghua University, Beijing, 100084 China

**Keywords:** *Apis mellifera*, *Apis cerana*, Microfluidic droplet, High-throughput cultivation, Gut microbiota, Strain diversity, Host specificity

## Abstract

**Background:**

Symbiotic gut microbes have a rich genomic and metabolic pool and are closely related to hosts’ health. Traditional sequencing profiling masks the genomic and phenotypic diversity among strains from the same species. Innovative droplet-based microfluidic cultivation may help to elucidate the inter-strain interactions. A limited number of bacterial phylotypes colonize the honeybee gut, while individual strains possess unique genomic potential and critical capabilities, which provides a particularly good model for strain-level analyses.

**Results:**

Here, we construct a droplet-based microfluidic platform and generated ~ 6 × 10^8^ droplets encapsulated with individual bacterial cells from the honeybee gut and cultivate in different media. Shotgun metagenomic analysis reveals significant changes in community structure after droplet-based cultivation, with certain species showing higher strain-level diversity than in gut samples. We obtain metagenome-assembled genomes, and comparative analysis reveal a potential novel cluster from *Bifidobacterium* in the honeybee. Interestingly, *Lactobacillus panisapium* strains obtained via droplet cultivation from *Apis mellifera* contain a unique set of genes encoding l-arabinofuranosidase, which is likely important for the survival of bacteria in competitive environments.

**Conclusions:**

By encapsulating single bacteria cells inside microfluidic droplets, we exclude potential interspecific competition for the enrichment of rare strains by shotgun sequencing at high resolution. The comparative genomic analysis reveals underlying mechanisms for host-specific adaptations, providing intriguing insights into microbe-microbe interactions. The current approach may facilitate the hunting for elusive bacteria and paves the way for large-scale studies of more complex animal microbial communities.

Video Abstract

**Supplementary Information:**

The online version contains supplementary material available at 10.1186/s40168-022-01333-9.

## Background

The animal gut is inhabited by billions of bacterial cells from a wide range of taxa with a rich genomic and metabolic pool. The gut microbiota can profoundly affect hosts’ physiology, metabolism, immunity, and behaviors [1]. Over the years, investigations based on marker gene amplification and shotgun metagenomic technologies have expanded our understanding of the diversity of the complex gut microbial communities and revealed intriguing associations with hosts’ health [2, 3]. However, a great deal of genomic and phenotypic diversity exist among strains of the same species, which causes a massive disconnect between the sequencing results and the actual existence of bacteria strains. Therefore, culture is vital for studies of the intestinal microbiome. By culturing specific bacterial individuals, we can recover the complete reference genome and accurately identify the taxonomic and functional potential of specific rare strains [4, 5]. However, traditional culture methods are often limited by substrates and growth conditions. Slow-growing bacteria present in low abundance are significantly affected by inter-species competition [6]. Thus, only a few have been effectively characterized [7]. In addition, most cultivation efforts relying on traditional strategies require manual selection of large numbers of colonies; the cost and throughput severely limit the exploration progress of rare microbial taxa [7].

To circumvent or minimize the potential limitations of traditional culture strategies, innovative technologies have broadened the toolkits for microbial isolation and cultivation. Droplet-based microfluidics is a novel technology for manipulating and processing small amounts of droplets carried by corresponding immiscible phases [8]. The effects of overgrown fast-growing populations in community culture can be eliminated by compartmentalizing microbes in droplets of media that are tens to hundreds of micrometers in diameter and separated by immiscible oils and engineered surfactants [9]. Since microfabricated physical pores or channels do not confine droplets, millions of independent culture systems can be created quickly. So far, platforms for high-throughput automated isolation, culture, and sorting of gut microbial members in microfluidic droplets have been developed and used for isolating microbes from seawater and soil communities [10, 11], high-coverage genome sequencing of single cells [12], and targeted screening of microbiome resource searching for probiotics and physiologically active compounds [13].

Animals carry complex communities of symbiotic microbiota that typically encompass strains with highly variable gene content and existence a rich strain-level diversity [14]. Among the human health conditions linked to microbial communities, phenotypes are often associated with only a subset of strains within causal microbial groups [15]. However, important strains are deficient in abundance and often require a high sequencing depth to be detected [16]. Their isolations are often affected by the competition from fast-growing individuals [7]. Therefore, it is promising to apply the innovative microfluidic droplet method to study the strain-level composition and functional diversity of gut microbiota. Combined with 16s rRNA amplicon sequencing, preculture of microfluidic droplets exhibited broad applicability for investigating the dietary carbohydrate metabolism [17] and antibiotic-resistance of intestinal bacteria [18].

Honeybees (*Apis mellifera*) are important pollinators of plants in both natural and agricultural landscapes [19]. Studies have shown that honeybee gut microbiota affects host nutrition, weight gain, and endocrine signaling [20]. Moreover, gut microbial members also have immunomodulation effects on bees [21] and protect honeybees from the opportunistic pathogens [22], which is critical to their survival and health in a complex environment [23]. Furthermore, there are known interaction between honeybee intestinal microorganisms and some honeybee diseases, for example, *Nosema ceranae* infection can promote proliferation of yeasts in honeybee gut and cause disease [24]. Therefore, intestinal bacteria are important for the bee hosts, and more investigations on the roles of different gut microbial members are needed. Compared with other animals, *A. mellifera* harbors a simple, recurring, and stable set of gut bacteria, including shared core phylotypes of *Gilliamella*, *Snodgrassella*, *Bifidobacterium*, *Lactobacillus* Firm4 and Firm5, and several host-specific phylotypes [25]. These bacteria are host-adapted, and each species cluster occupies particular niches and spatial locations in the host [21]. Although closely related bacterial species co-colonize stably in the bee gut, competitions exist between species. For example, food polysaccharide is an important factor in determining the coexistence state of *Lactobacillus* species in the gut of honeybee [26]. While the honeybee gut is composed of a limited number of bacterial phylotypes, metagenomic and single-cell genomic analyses revealed significant strain-level diversity [27, 28]. Moreover, individual strains with unique genomic potentials possess different capabilities, which are functionally relevant to hosts' nutrition metabolism and health [29, 30].

In this study, we first constructed a microfluidic droplets platform and generated droplets encapsulated with individual bacterial cells from the honeybee gut. Subsequently, we performed incubation and determined the growability of microorganisms within microfluidic droplets. To demonstrate the utility of our platform for measuring strain-level diversities, we employed metagenomic sequencing and obtained potentially novel strains from different bacteria genera. The comparative genomic analysis of *Lactobacillus panisapium* found that strains from *A. mellifera* contain a set of genes encoding arabinofuranosidase, which is likely important for the survival of bacteria in competitive environments.

## Methods

### Honeybee gut sample collection

The honeybees (*A. mellifera*) used in this study were obtained from an apiary in Kunming, Yunnan Province, China, and all individuals were hive bees. Newborn adult bees were collected from one single frame ~ 10 days after emergence. As described in previous studies [31], the entire guts were aseptically dissected by gently pulling the strings without touching the abdomen surface using sterilized forceps. Subsequently, dissected guts were directly crushed in 25% (*v*/*v*) glycerol using an electric tissue grinder (OSE-Y30; Tiangen Biotech Co., Ltd., Beijing, China). We obtained intestinal samples from a total of 30 individual bees, and the dissected guts were pooled and thoroughly mixed. Then the gut homogenate was aliquoted and stored at – 80 °C for subsequent droplet generation processes.

### Microfluidic droplets generation

Droplets were made on a droplet entrapping microfluidic cell-sorter (DREM cell; Yuanqing Tianmu Biotechnology, Ltd., Wuxi, Jiangsu, China). The microfluidic chip was designed by AutoCAD and manufactured via soft lithography (Additional file 1: Figure S1). The negative photoresist SU-8 2015 (Westborough, MA, USA) was rotated and coated on a 4-inch silicon wafer to obtain the 15 μm height channel. The Polydimethylsiloxane (Midland, MI, USA) prepolymer was mixed with the curing agent and poured onto the silicon mold. After removing bubbles, it was heated overnight at 65 ^o^C. The microchannel pattern was peeled off from the silicon mold and punched at a set position to form an inlet and outlet for the sample and reagent. The chips and glass slides were exposed to 140 W of oxygen plasma (PDC002; Harrick Plasma, Ithaca, NY, USA) for 60 s and heated for 24 h at 120 ^o^C. The chip consists of two inlets for the continuous oil phase and aqueous phase, respectively, and one outlet for collecting highly monodisperse water-in-oil emulsions. On the chip, the two liquids confined within microfluidic channels are brought together using pressure pumps, followed by the subsequent formation of droplets at the flow-focusing junction due to shear stress.

To guide the subsequent microfluidic droplet generation process, we first roughly estimated the bacterial concentration of the mixed intestinal sample. It was diluted and plating on Brain Heart Infusion (BHI; Oxoid, Basingstoke, UK) supplemented with 5% (*v/v*) defibrinated sheep blood (Solarbio, Beijing, China) at 35 °C under a CO_2_-enriched atmosphere (5%) for 2 days for plate counting, and the concentration of the sample was about 10^8^ CFU/mL. The droplet generation oil for EvaGreen® (Bio-Rad Laboratories, Inc., Hercules, CA, USA) was used for the continuous oil phase. According to the Poisson distribution *P*(*X* = *n*) = *ⅇ*^−*λ*^(*λ*^*n*^ ∕ *n*!) (Additional file 1: Figure S2), the droplet occupancy (*n*) is related to the average number of cells per droplet (*λ*) given by the equation *λ* = *ρV*, where *V* is droplet volume, and *ρ* is cell density. In this study, assays were performed using *λ* values of 0.3 to minimize the number of droplets loaded with two more bacterial cells; therefore, for a given droplet volume, there is only one corresponding suspension concentration (Additional file 1: Table S1). In this study, two pressure pumps were used to control the oil and cell suspension flow rates, and we controlled the droplet volume by adjusting the flow rate ratio to a diameter of 30 μm (~ 14 pL). Therefore, the frozen intestinal samples were centrifuged (5000×*g*, 5 min), washed, and resuspended in BHIB (CM1135, Oxoid Ltd., Basingstoke, UK) and MRS broth (CM1175, Oxoid Ltd., Basingstoke, UK) respectively and diluted to ~ 2 × 10^7^ CFU/mL for droplets generation. In addition, the suspension was incubated with 1 μg/mL DAPI stain solution (E607303; Sangon Biotech Co., Ltd., Shanghai, China) at 35 ^o^C for droplet generation. Then, the droplets were introduced into a hemocytometer plate and then visualized by an inverted fluorescence microscope (Eclipse Ts2; Nikon Instech Co., Ltd., Tokyo, Japan) to test whether the distribution of bacteria was consistent with the theoretical calculation under these parameters. For the incubation of the droplets, ~ 3 mL of the emulsion was loaded into Teflon tubes (about 7.7 m, φ = 0.71 mm; Suzhou Volsun Electronics Technology, Suzhou, China) to maintain stability during the incubation process. Three samples were generated separately for each type of medium, each containing around 2 × 10^8^ droplets. All samples were incubated for 120 h at 35 °C under a CO_2_-enriched atmosphere (5%). After incubation, the droplets were introduced into a cell counting chamber slide (Bodboge, Shenzhen, China) for observation. The inverted fluorescence microscope (Nikon) was also used to examine the stability of microfluidic droplets and microbial growth.

### Droplet DNA extraction and shotgun metagenomic sequencing

After cultivation, we pipetted out all the droplet emulsion using sterile syringes, mixed it with an equal volume of 1H,1H,2H,2H-Perfluoro-1-octanol (PFO; CAS647-42-7, Shanghai Aladdin Bio-Chem Technology Co., Ltd., Shanghai, China), vortexed and centrifuged the solution, and removed the oil and PFO carefully. The Ezup Column Bacteria Genomic DNA Purification Kit (Sangon Biotech Co., Ltd., Shanghai, China) was used for DNA exaction. The gut DNA was extracted using the cetyltrimethyl ammonium bromide (CTAB) buffer method [33]. All DNA samples were submitted to the Novogene Company (Beijing, China) for shotgun metagenome sequencing. NEBNext UltraTM II DNA Library Prep Kit for Illumina (New England Biolabs, MA, USA) was used for the generation of sequencing libraries, and Qubit 3.0 Fluorometer (Life Technologies, Grand Island, NY, USA) and Agilent 4200 (Agilent, Santa Clara, CA, USA) system were used for library quality assessment. The libraries were then sequenced on the Illumina HiSeq platform with 150-bp paired-end reads.

### Species- and strain-level community profiling

After the sequencing results were obtained, fastp [32] was used for adaptor trimming and quality control of the raw sequencing data. Then reads are aligned to the genome of *A. mellifera* (GCA_003254395), and any *A. mellifera* reads were removed from the metagenomic data. The species- and strain-level community profiling and gene content estimation were performed using the Metagenomic Intra-Species Diversity Analysis System (MIDAS) pipeline [34]. As described in our previous study [35], the custom database included genomes of pure isolates from the guts of *A. mellifera*, *Apis cerana*, *Apis dorsata*, and Bombus species. The relative abundance of species clusters was estimated by mapping quality-filtered reads to the database of phylogenetic marker genes using HS-BLASTN with the “run_midas.py species” module. Then, the results across all samples were combined using “merge_midas.py species”.

We subsequently used the “run_midas.py snps” module of the MIDAS pipeline to profile single nucleotide polymorphisms (SNPs) by identifying single nucleotide variants (SNVs) diversity for each species cluster in metagenomic samples. The genomes with the highest completeness and lowest contamination were selected as representatives for each species cluster, and our metagenomic reads were aligned to the reference genomes using Bowtie2 [36]. Pileups of each sample were generated using SAMtools [37], and the nucleotide variation statistics were then counted at each genomic site. The results were merged using the “merge_midas.py snps” module to generate core genomic SNP matrices to compare nucleotide variants in genomic loci and metagenomic samples present in different cultured samples. We focused on the bi-allelic SNVs prevalent in more than 5% of the samples. Strain-level diversity within species clusters in each sample was estimated by quantifying the fraction of SNVs in protein-coding genes (number of SNVs/length of genes). We also generated a Jaccard distance matrix based on shared polymorphic sites and performed principal coordinate analysis and visualization based on the pairwise fractions of shared SNVs for different samples using the vegan package [38].

### Metagenome binning and functional annotation of MAGs

Metagenomic binning was performed using the metaWRAP pipeline [39]. Following de novo assembly with the metaSPAdes [40], the quality-controlled reads (about 30 million reads per sample) were mapped to the assembled contigs using Bowtie2 to generate a coverage score for individual contigs. The metagenome-assembled genomes (MAGs) were recovered from each sample independently using three different tools: CONCOCT, MaxBin, and metaBAT. Subsequently, the three final bin sets produced were consolidated into a single and more robust bin set with the minimum completion (-c 50) and maximum contamination (-x 30) parameters using the “Bin_refinement” module in metaWRAP. The completeness and contamination of each MAG were estimated using CheckM software [41], and each bin was taxonomically assigned against the Genome Taxonomy Database with GTDB-tk [42]. To get a complete picture of the exact distribution of microbial members in the samples, we did not perform de-duplication. In a further study, phylogenetic analyses of genomes were conducted with the PhyloPhlAn 3.0 under the “--diversity low” parameter [43], and the iTOL web-based software [44] was used for the visualization of phylogenetic trees. Whole-genome average nucleotide identity (ANI) was calculated using the web service JSpeciesWS [45]. After protein-coding gene prediction using Prodigal software [46], the grouped amino acid sequences were submitted separately to the Orthovenn2 tool web server [47] for orthologous analysis to compare and annotate the orthologous cluster between genomes under the parameters of “*E*-value 10^−15^” and “Inflation value 1.0”. For *Lactobacillus* Firm5, after identifying unique gene clusters, cblaster software [48] was used to search and visualize for collocated protein-coding regions locally within our Firm5 database. We also performed carbohydrate-active enzymes (CAZymes) annotation of genomes against the dbCAN2 database using HMM search approach as reported by Zhang et al. [49]. All heatmaps were visualized by the pheatmap package [50] in R software.

## Results and discussion

### Microfluidic single-cell encapsulation and cultivation of honeybee gut microbiota

**Fig. 1:**
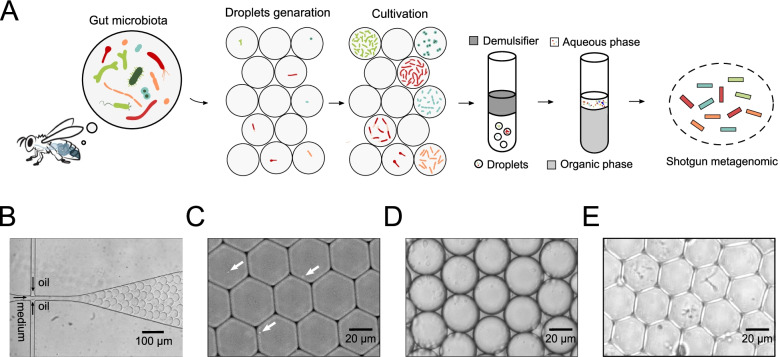
Single-cell encapsulation and cultivation of honeybee gut bacteria in microfluidic droplets. **A** Principal scheme for single-cell encapsulation and cultivation of honeybee gut bacteria using the microfluidic droplet platform. After isolating individual bacteria in a single droplet, the emulsifier was added to break up droplets. The bacteria in the upper aqueous phase were collected for shotgun metagenomic sequencing. **B** Single bacteria cells are isolated in droplets containing culture medium, and the emulsion was incubated for 120 h. (see also Additional file 2: Video S1). **C** The distribution of bacteria cells in the droplets was visualized by fluorescence microscopy. White arrowheads point toward individual bacterial cells. **D** After 120 h, the microfluidic droplets remain stable during cultivation. **E** Distinct morphologies across droplets of cultivated bacteria. (See also Additional file 3: Video S2)

To isolate and culture individual bacterial cells from *A. mellifera* microbiota communities, an array of high-throughput droplet microfluidic technologies was developed (Fig. 1A). We generated microfluidic droplets of bacterial cells by integrating the commercial droplet entrapping microfluidic cell-sorter (DREM cell) with two pressure pumps and a high frame rate camera (Additional file 1: Figure S1). We selected MRS and BHI as the liquid medium because they are the most commonly used culture media for the gut bacteria of honey bees [51]. We mainly used the classic continuous droplet generation technique named “flow-focusing” [52]. The droplets were generated at the flow-focusing junction from the liquid culture medium into oil (Fig. 1B, Additional file 2: Video S1). The number of cells contained in the formed droplet is determined by the probability that a given volume of initial cell suspension contains a given number of cells. It follows a Poisson distribution (Additional file 1: Figure S2, Table S1). Thus, the frozen intestinal suspensions were resuspended and diluted to 2 × 10^7^ CFU/mL for droplets generation so that, in principle, ~ 22% of encapsulated microbial cells in the droplets (~ 14 pL) initially contain only one cell, and less than 5% of droplets contain two or more live bacterial cells stochastically. To ensure the single-cell deposition per droplet, we encapsulated DAPI-stained bacteria cells under the same parameters. The distribution of bacteria cells in the droplets was visualized using fluorescence microscopy (Fig. 1C). We found that most of the droplets encapsulated with bacteria contained only one cell, and the proportion of droplets with more than two cells was negligible. Notably, due to the random distribution of bacteria under current droplet generation methods, ~ 70% of the droplets contained no bacterial cells. Such a distribution of bacterial cells is likewise an obstacle to the production of single-cell droplets [53]. To obtain pure populations of positive droplets, the generation of droplets is always coupled with detection and sorting by flow-cytometry technologies [54]. Here, we did not sort bacteria to track single bacterial cells but performed shotgun metagenomes for the whole community to secure all genomic diversity. We generated approximately 2 × 10^8^ droplets for each sample, which means that more than 4 × 10^7^ droplets only contain individual bacteria, sufficient for the subsequent sequencing analysis.

The generated droplets were then collected in a Teflon tube (Additional file 1: Figure S3) and incubated for 120 h at 35 °C. Since most honey bee intestinal bacterial members can only grow in an elevated CO_2_ environment [51], the droplets were incubated under a CO_2_-enriched atmosphere (5%). The droplets remained stable for several days in culture, and the diameter of the droplet did not change significantly (Fig. 1D). After 120 h of cultivation, microscopy showed the presence of live bacteria moving in the droplet (Fig. 1E, Additional file 3: Video S2), suggesting that a single bacterial cell could reproduce in the droplets. The consistent morphology of the bacteria in each droplet indicated that isolated viable strains could clonally replicate within a droplet. Moreover, the living cells from individual droplets showed different morphologies, indicating the segregation of diverse clonal populations from the microbial communities. Thus, our platform encapsulated individual honeybee gut members into microfluidic droplets, and the droplet environment could support the growth and metabolism of microorganisms. Ecological competition is prevalent in natural gut communities, and bacteria compete for space and resources [55]. In the traditional culture process, certain microorganisms quickly dominate the culture system and prevent the growth of others using the same substrate but with a lower affinity [7]. The droplet-based cultivation isolated bacteria within an enclosed single droplet. The slow-growing bacteria avoid the competitive overgrowth, providing an opportunity to cultivate slow-growing microorganisms. However, many microorganisms rely on products of syntrophic partners and the accumulation of signals for quorum sensing. Thus, the microfluidic chips that grow bacteria are fully sealed chambers, likely prohibiting the cultivation of dependants [7].

After incubation, we pipetted out the droplet emulsion, mixed it with an equal amount of emulsifier (PFO), vortexed and centrifuged the solution, removed the organic phase (oil and PFO) located in the lower phase, and collected the bacteria in the upper aqueous phase for DNA extraction (Fig. 1A). In addition, the DNA extracted from preculture intestinal homogenates was also submitted for sequencing.

### Community structure after microfluidic droplet cultivation

**Fig. 2:**
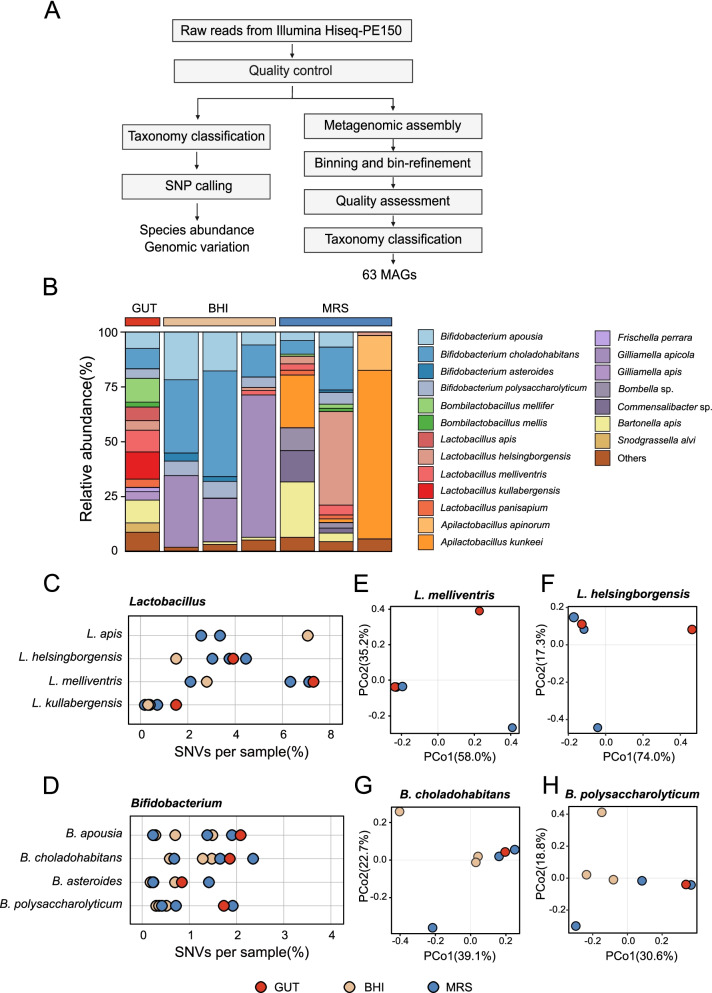
Strain-level compositions of honeybee gut shift after droplet-based cultivation. **A** Schematic of the data analysis workflow. After base quality control, the bacterial species abundance and strain-level genomic variation were estimated using the MIDAS pipeline, and metagenomic binning was performed to restore the metagenome-assembled genomes. **B** Species-level profiles for the gut sample (GUT) and the picodroplet samples using the Brain Heart Infusion (BHI) or the MRS broth (MRS) after cultivation. **C**, **D** Fraction of single-nucleotide variants (SNVs) within core genes in each sample for *Lactobacillus* (**C**) and *Bifidobacterium* (**D**). **E**–**H** Principal coordinate analysis plots based on the pairwise fractions of shared SNVs (Jaccard distance) for the species *Lactobacillus melliventris* (**E**), *Lactobacillus helsingborgensis* (**F**), *Bifidobacterium choladohabitans* (**G**), and *Bifidobacterium polysaccharolyticum* (**H**). Dots represent individual samples, color-coded by the medium used for cultivation

Approximately 30 million pair-end reads (150 bp) per sample were produced using shotgun metagenomic sequencing technology. After base quality control, reads were aligned to the genome of the host, and ~ 7% of reads derived from the bee host were removed. For samples after microfluidic-droplets incubation, almost all reads were from bacteria without host contamination. Our data analysis consists of two major steps (Fig. 2A). We first estimated bacterial species abundance and strain-level genomic variation, including SNPs from shotgun metagenomic reads, using the MIDAS pipeline with a custom database for bee microbiome [35]. Then, we restored the bacterial genomes using genome-resolved metagenomic approaches based on the metagenomic assembly and clustering of contigs through the metagenomic binning procedure.

The results of the MIDAS pipeline show that the uncultured gut community samples were dominated by five core bee gut members, and most of the common gut microbial species in *A. mellifera* could be detected (Fig. 2B). However, after our droplet-based cultivation, genus-level community compositions changed significantly, and the results differed depending on the medium (Fig. 2B). *Bifidobacterium* and *Gilliamella* were significantly enriched after 120-h incubation in BHI-droplets, while *Lactobacillus* and *Apilactobacillus* were enriched within the MRS-cultured groups. We then focused on species-level changes in these genera. After cultivation, the alpha diversities of communities from each sample were significantly reduced (Additional file 1: Figure S4). Principal coordinates analysis (PCoA) revealed distinct clustering of microbiota composition for the different medium groups (Additional file 1: Figure S4). Further, the relative abundance of specific species was significantly increased after cultivation, such as *Bifidobacterium apousia* and *Bifidobacterium choladohabitans* of the BHI-cultured group. Likewise, *Apilactobacillus kunkeei* and *Lactobacillus helsingborgensis* were accumulated in MRS-droplets, and some rare species (< 0.01% relative abundance) in the community have been enriched to higher abundance in droplets, such as *Gilliamella apicola* (> 19% in BHI droplets), suggesting that they may be more adapted to the incubation conditions. Correspondingly, some species may not grow or grow slowly in the droplets, exhibiting a decrease in relative abundance during cultivation. Therefore, droplet-based culture reduced the species-level diversities compared to the original honeybee gut communities, consistent with previous studies [18].

Since it has been suggested that the high strain-level diversities from *A. mellifera* species, especially for *Lactobacillus* [56] and *Bifidobacterium* [57], we compared the strain-level genomic variation by calculating the fraction of single nucleotide variants (SNVs) sites among all profiled sites for each species (Fig. 2C, D). We first focused on *Lactobacillus* species: *Lactobacillus apis*, *L. helsingborgensis*, and *Lactobacillus melliventris* harbor more than 2% SNVs in most samples, while the *Lactobacillus kullabergensis* showed a lower level of variations than other *Lactobacillus* species. In addition, we observed a higher proportion of SNVs for *L. apis* in the BHI-cultured group. In MRS-cultured groups, *L. helsingborgensis* and *L. melliventris* were detected with more single-nucleotide variants, indicating the adaptability of the medium varies significantly among different strains. As for *Bifidobacterium*, there were few differences between different species from droplets-cultured groups; nearly all the bacterial species had less than 2.5% SNVs. Interestingly, we found that for *L. kullabergensis* and *L. melliventris* in MRS-cultured groups, *Bifidobacterium coryneforme*, *B. choladohabitans*, and *Bifidobacterium polysaccharolyticum* in BHI groups, the fractions of SNVs were even higher than the gut samples. This implied that some rare strains were enriched by droplet-based cultivations, and they were not detected by the previous depth of sequencing. To visualize the distribution of SNVs across samples, we calculated Jaccard distances between all pairs of samples based on shared SNVs. PCoA revealed that the composition of SNVs was significantly different among the same species under different culture conditions, illustrating that distinctly different strains were specifically enriched under different culture conditions (Fig. 2E–H).

In summary, we obtained multiple morphologies of microorganisms through our high throughput microfluidic droplets cultivation. Despite the number of species being close to the uncultured group, the strain-level variations differed among samples, demonstrating that our culture strategy did enrich different individual bacterial cells of honeybee gut communities in a single droplet. For some species, we even could observe higher strain-level diversities after droplet cultivation relative to uncultured samples, demonstrating the potential of our platform for the isolation and enrichment of rare microbial strains. These strains are often challenging to detect and quantify because of their low abundance in natural communities, and high sequencing depth is essential for their investigation.

### Sixty-three draft MAGs were recovered after microfluidic droplet cultivation

**Fig. 3:**
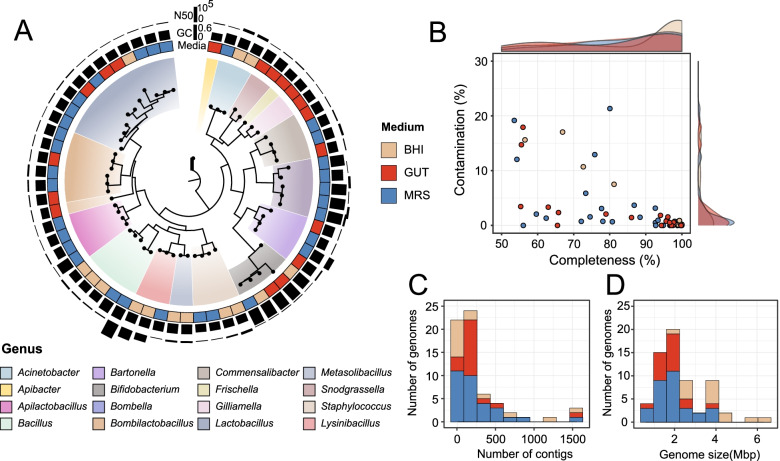
Metagenome-assembled genomes (MAGs) recovered from honeybee gut communities after droplet-based cultivation. **A** A microbial phylogeny of 63 MAGs. Concentric rings moving outward from the tree show the type of medium, GC content, and N50, respectively. See also Additional file 4: Dataset S1. (**B**) The completeness and contamination estimations for the MAGs. Dots represent individual MAGs. **C**, **D** The frequency distribution of the number of contigs (**C**) and genome sizes (**D**) of MAGs

**Fig. 4:**
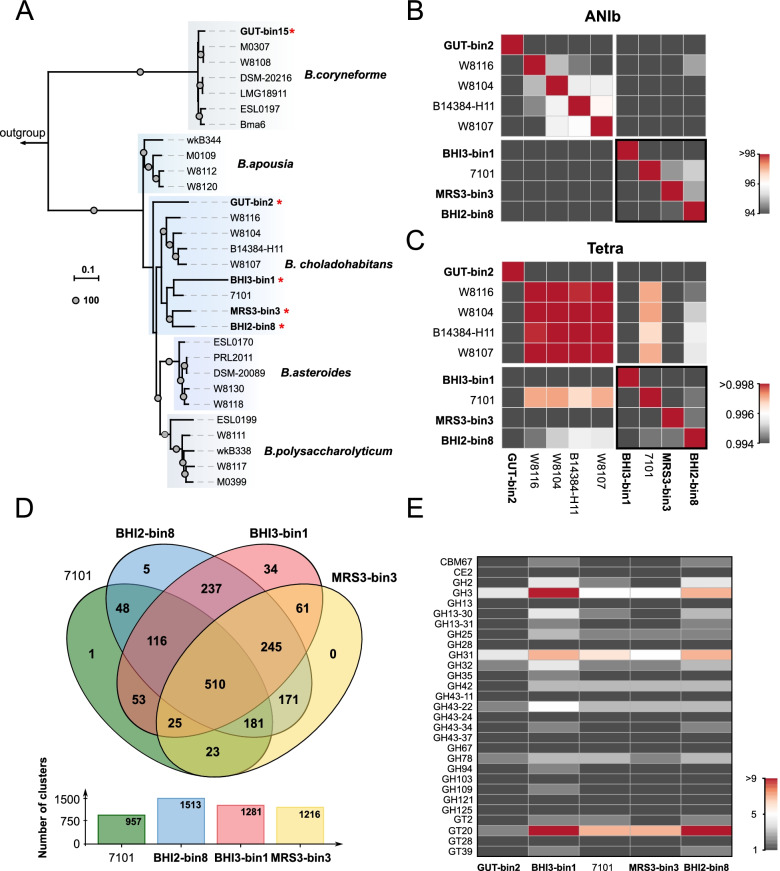
Comparative analysis revealed the genomic diversity of *Bifidobacterium* in the honeybee gut. **A** Whole-genome phylogenetic tree based on five MAGs and representative isolates' genomes of *Bifidobacterium*. The tree was rooted with the sequence of *Bifidobacterium tissieri* DSM 100201. Only bootstrap values of 100% are shown at node points. **B**, **C** Heatmaps show the values of pairwise ANIb (**B**) and TETRA (**C**) between nine genomes from *Bifidobacterium choladohabitans*. **D** Venn diagram of the orthologous gene clusters. See also Additional file 5: Dataset S2. **E** Distribution of CAZyme genes in the *Bifidobacterium* genomes

**Fig. 5:**
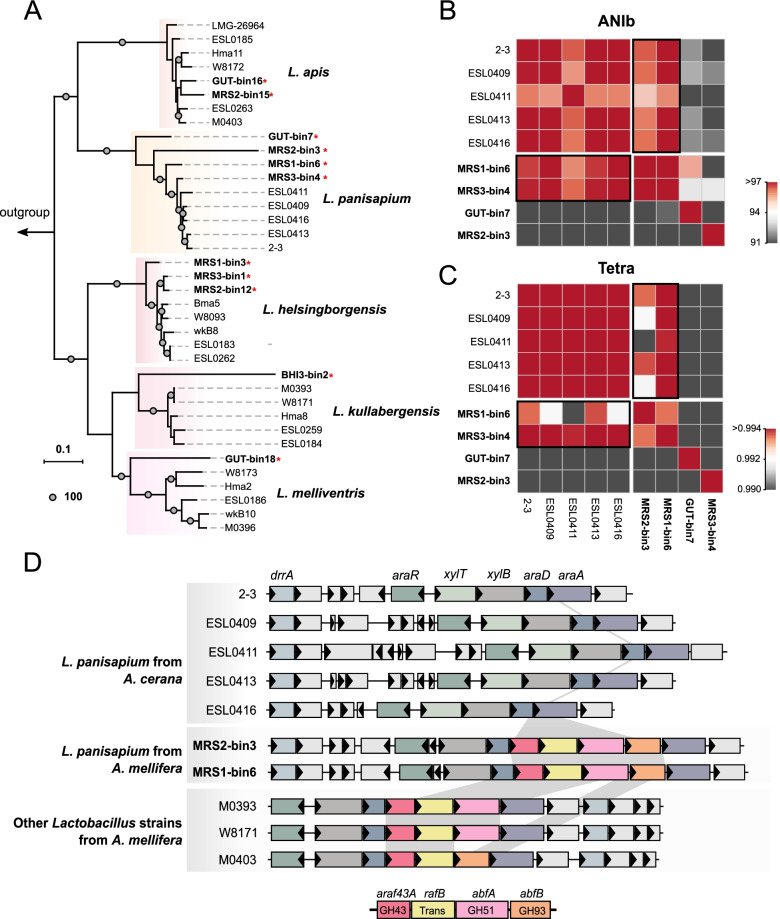
Polysaccharide degradation genes are potentially important for the host-specific adaptation of *L. panisapium* in *A. mellifera*. **A** Whole-genome phylogenetic tree based on 11 MAGs and representative isolates' genomes of *Lactobacillus* Firm5. The tree was rooted with the sequence of *Lactobacillus terrae* NIBRBAC000499792^T^. Only bootstrap values of 100% are shown at node points. **B**, **C** Heatmaps show the values of pairwise ANIb (**B**) and TETRA (**C**) between nine genomes from *Lactobacillus panisapium*. **D** Genomic region encoding exo-alpha-(1-> 5)-l-arabinofuranosidase genes in *L. panisapium* strains. Vertical grey blocks connect the homologous genes

Due to some rare strains being detected in our droplets-cultured samples, de novo assembly and binning were used on shotgun metagenomic reads. Metagenomes were assembled independently to reduce the influence of strain variation and improve the recovery of closely related genomes [58]. Sixty-three MAGs were identified after refinement and filtering of the resulting population genomes (Fig. 3A, Additional file 4: Dataset S1), and we evaluated their quality (Fig. 3B). About 55 MAGs had completion scores above 60%, and almost all had less than 20% genomic contamination. Further, recovered MAGs had a median genome size of 1.9 Mbp, 133 contigs, and a median N50 of 20.6 kbp (Fig. 3C, D, Additional file 4: Dataset S1). Taxonomic annotation using GTDB indicated the majority of our MAGs belonged to phyla *Proteobacteria* and *Firmicutes* (Fig. 3A, Additional file 4: Dataset S1). We also obtained five bacterial genomes from *Actinobacteria*, all of which were *Bifidobacterium*. To further characterize assembled genomes, we classified MAGs and reconstructed genomic phylogenetic trees with genomes from our database (Fig. 4A, Fig. 5A, Additional file 1: Figure S5–S9).

### Investigation of samples after droplet-based cultivation revealed the diversity of *Bifidobacterium* in honeybee gut

*Bifidobacterium* spp. is one of the core gut members of *A. mellifera*. So far, five species (*B. coryneforme*, *B. apousia*, *B. choladohabitans*, *Bifidobacterium asteroides*, and *B. polysaccharolyticum*) have been isolated and characterized from *A. mellifera* [57]. However, based on the phylogenetic tree (Fig. 4A), all four MAGs from droplets-cultured samples were clustered with *B. choladohabitans*. Moreover, we noted that three MAGs were clustered with the strain of *B. choladohabitans* 7101, implying the existence of potential novel cluster species. To quantify the magnitude of differences between genomes, we evaluated the Overall Genome Relatedness Index based on average nucleotide identity (ANI) analysis (ANIb based on BLASTN) and tetranucleotide usage patterns. The ANIb values between strains from different clusters ranged from 89 to 94% (Fig. 4B). The pairwise comparisons are almost > 95% within one cluster (W8116, W8104, B14384-H11, and W8107), indicating that this cluster represents a species-level taxon. The TETRA values also illustrated this point (Fig. 4C). However, for another cluster mainly consisted of our MAGs (BHI3-bin1, 7101, MRS3-bin3, and BHI2-bin8), the pairwise comparisons were between 92 and 95%, such findings implied the genomic diversity of these strains. Indeed, Ellegaard et al. [59] examined the density distribution of similarity within this specific SDP from *Bifidobacterium*. They found a significant percentage of ANI values between 90 and 95% and speculated that additional equally divergent strains are presented in the bee gut community. Based on this, four different bifidobacterial species within this SDP were identified and characterized [57]. But we found a new cluster within the discontinuous regions, and the genomes of this cluster had almost all paired ANI values <95% nucleotide sequence identity for species by Richter and Rosselló-Móra [60], which further demonstrated the diversity of *Bifidobacteria* in the honeybee gut, and suggested the presence of species not previously detected by conventional methods.

To gain further insights into the differences between our MAGs and *B. choladohabitans* 7101, we used OrthoVenn2 [47] to identify orthologous genes among our cluster (Fig. 4D). The four strains of *Bifidobacterium* possessed 1710 gene families. In contrast, a core genome comprised 510 clusters of orthologous (Additional file 5: Dataset S2), only accounting for 29.8% of all gene families, indicating that there are apparent genetic differences between all these strains. Most of the annotation functions of core homologous clusters were involved in metabolic process, biological process, cellular metabolic process, hydrolase activity, and molecular function, which may be closely related to the survival of these strains.

Remarkably, the number of unshared clusters of the MAG "BHI3-bin1" was higher than related species. The subsequent GO enrichment analysis showed that six gene clusters were related to transmembrane transport (GO: 0055085) and fatty acid biosynthetic process (GO: 0006633) (Additional file 5: Dataset S2). These clusters encode multiple sugar-binding transport system permease protein (MsmG) and 3-oxoacyl-ACP synthase II (FabF), which were associated with the protein-dependent transport system responsible for the uptake of melibiose and raffinose. This implied the unique advantages of carbohydrate transport and fatty acid synthesis compared with the other strains.

In addition, we compared MAGs from the different culture mediums, and the two BHI-associated MAGs shared 237 unique homologous gene clusters (Additional file 5: Dataset S2). GO analysis indicated that 16 were related to transmembrane transport, including formylaminopyrimidine transport permease ThiX, polygalacturonan/rhamnogalacturonan transport system permease YtcP, and putative ABC transporter permease ORF2. In addition, seven gene clusters were related to the inositol catabolic process (GO:0019310), encoding inositol 2-dehydrogenase, 3D-(3,5/4)-trihydroxycyclohexane-1,2-dione hydrolase, and inosose dehydratase. Interestingly, inositol utilization was reported as part of cell mass generation of *Corynebacterium glutamicum* during growth on the BHI [61].

The members of *Bifidobacterium* have been identified as the key polysaccharide degrader in the bee gut community [30]. However, it has been reported that strains from different phylogenetic clusters vary in the CAZyme repertoires for hemicellulose metabolism [57]. Therefore, we comprehensively analyzed the composition of CAZyme genes in our assembly genomes. Generally, numerous carbohydrate-binding modules (CBMs), glycoside hydrolases (GHs), carbohydrate esterases (CEs), and glycosyltransferases (GTs) were identified in all genomes. The genomes from the same cluster possessed similar CAZyme profiles (Fig. 4E), which agrees with the previous genomic study [30]. We further focused on the suspected novel clusters. MAGs from BHI post-culture samples tended to have more GH3, GH31, and GT20, revealing the different performances of some bifidobacterial strains in polysaccharide degradation.

### Polysaccharide utilization is important for the survival of *L. panisapium* in *A. mellifera*

*Lactobacillus* Firm5 is the most widely distributed and abundant phylotype in the bee gut microbiota [62]. Four deep-branching species of Firm5 have been identified in the gut of *A. mellifera*, with pairwise average ANI values below 90% [59, 63]. Here, we obtained 11 MAGs from Firm5, seven of which were derived from the samples of MRS-droplet cultivation. Unlike *Bifidobacterium*, the assembled MAGs are distributed in different species clusters (Fig. 5A). Specially, we obtained MAGs of *L. helsingborgensis* from three different MRS replicating samples, while only one MAG of *L. kullabergensis* was from the BHI sample. Notably, three MAGs from the MRS group and one assembly from the gut sample fell into the *L. panisapium* cluster, with five isolates from *A. cerana* [62]. To examine the genomic similarities, we compared the intra-species genomic variation of *L. panisapium* by pairwise comparison of ANIb and Tetra values (Fig. 5B, C). Except for one genome from the gut sample (GUT-bin7) and one genome (MRS3-bin4) with relatively low completeness (53.53%), two MAGs obtained from the MRS-droplet cultivation showed high similarity to the other strains from *A. cerana* (> 95% for ANIb, > 0.991 for Tetra).

Remarkable strain-level diversities were observed within *Lactobacillus* Firm5, and the genomic variation was associated with the functions in carbohydrate metabolism [56]. Thus, we compared the homologous gene clusters of different strains. Interestingly, genes specific to *A. mellifera* strains were related to the l-arabinose metabolic process (GO:0046373; GO:0019569) (Additional file 6: Dataset S3), including genes of *araf43A* (extracellular exo-alpha-(1-> 5)-l-arabinofuranosidase), *rafB* (raffinose permease), *abfA* (intracellular exo-alpha-(1-> 5)-l-arabinofuranosidase), and *abfB* (extracellular exo-alpha-l-arabinofuranosidase). These genes were explicitly inserted between *araA* and *araD*, which are part of the l-arabinose operon [65], forming a co-locate gene locus with *araR* (LacI-type transcriptional regulator), *xylT* (xylose transporter), and *xylB* (xylulokinase). However, these genes were totally absent in strains from *A. cerana*, while other monosaccharide degradation genes showed synteny (Fig. 5D). Moreover, the gene set of l-arabinofuranosidase was present in other Firm5 species from western honeybees, which implied that they were important for the colonization of *A. mellifera*.

*L. panisapium* was first identified from the honey bread of *A. cerana* [66] and formed monophyletic clusters from other species, suggesting that it might be a specific species to *A. cerana*. Although trace amounts of shotgun metagenomic reads from *L. panisapium* were detected in gut samples from *A. mellifera*, it was hypothesized to be the bacteria transfer due to contact with non-native symbionts [62]. Here, we obtained bacterial genomes that showed high similarity with the known *L. panisapium* strains from *A. mellifera*. However, significant variation was identified between genomes from different bee hosts, suggesting that these *L. panisapium* strains enriched by microfluidic droplets were native to *A. mellifera* rather than transfer or contamination. Our microfluidic droplet cultivation strategy enriched the low-abundance bacteria, probably because of the exclusion of their competition with other high abundant strains.

The honeybee diet has various polysaccharide components, and intestinal bacteria are the main agents in the degradation of these polysaccharides [30]. *Lactobacillus* Firm5 are significant fermenters of dietary carbohydrates for bees [56]. However, gut bacteria of bees have obvious distinct repertoires of carbohydrate-active enzymes and occupy different glycan niches. For Firm5, Brochet et al. [67] demonstrated that polysaccharide fractions are the main determinants for the structure of different species. We showed that *L. panisapium* strains from *A. mellifera* contain unique genes of polysaccharide metabolism, specifically for the hydrolysis of arabinoxylans to oligosaccharides. Moreover, these genes cluster with monosaccharide metabolism genes, forming CAZyme gene clusters. Similar structures have been found in *Bifidobacterium* from *A. mellifera* [30]. Although *A. mellifera* and *A. cerana* have a similar dietary regimes, there may be differences in the specific composition of their diets [64]. *L. panisapium* in *A. mellifera* possibly compete to utilize polysaccharides from the host's diet, which confers a selective advantage for colonization.

Gut ecosystems often contain strains with highly variable genetic contents, and the strain-level diversity is substantial in host-associated bacterial communities [68]. Genomic analysis has shown that strain-level variants within microbial species are essential in determining functional capacities in honeybees [29, 30], which is also documented for humans [69]. It is crucial to identify and assess the strains with distinct genetic repertoires, probably affecting the establishment of a healthy symbiosis and host biology [70]. However, the gut microbial community is often complex, requiring a high sequencing depth to achieve a satisfactory resolution [16]. Through the microfluidic droplet cultivation platform, we were able to identify rare strains. Separate encapsulation excludes the effect of microbial competition during the culture process, allowing for the enrichment of strains that are hardly detected. Inevitably, certain strains may not be cultured due to the absence of co-dependent individual or population sensing signals. Overall, we established a microfluidic cultivation strategy combined with metagenomic analysis for honeybee gut symbionts, encouraging potential applications in other complex microbiota communities.

## Conclusions

In this study, we established a droplet microfluidic platform for the high-throughput culture of honeybee gut bacteria combined with shotgun metagenomic and binning strategy. Individual droplet encapsulation excludes the effect of competition during cultivation. However, it should be noted that the cultivation output would be modulated due to the selected culture medium. Strain-level analysis revealed potential novel species from the honeybee. In particular, a comparative analysis of *L. panisapium* found that strains from *A. mellifera* contain a set of genes related to the utilization of diet polysaccharides, which is likely important in competitive environments. In addition, cultures of Eukaryotes from the gut, such as fungi, will likely be studied in the future by adjusting droplet size, culture conditions, and sequencing approaches, which will further expand our understanding of the complex members of the gut. Overall, our results demonstrate the adaptability of droplet-based cultivation in investigating microbial diversity in the honeybee gut. This approach is also applicable for other complex communities, which may validate functional analyses predicted by the genomes.

## Supplementary Information

**Additional file 1: Figure S1.** Devices for microfluidic droplet generation. The microfluidic droplets of bacterial cells were generated by the droplet entrapping microfluidic cell-sorter integrating with two pressure pumps. The chip consists of two inlets for continuous and aqueous phases and one outlet for collecting the water-in-oil emulsions. The two liquids confined within microfluidic channels are brought together at the flow-focusing junction, and the generated droplets were collected in a Teflon tube. **Figure S2.** Poisson distribution curve. When the average number of cells per droplet (λ) is determined, the distribution of the number of bacteria in the droplet conforms to the Poisson distribution. **Figure S3.** The generated droplets are introduced into Teflon tubes for incubation. **Figure S4.** (A-B) The Shannon (A) and Simpson (B) diversity metrics of the data sets. (C-D) Microbiome compositions in different developmental stages are plotted on Jaccard (C) and Bray-Curtis (D) PCoA graphs. **Figure S5.** Whole-genome phylogenetic tree based on MAGs and representative isolates' genomes from *Apibacter.* **Figure S6.** Whole-genome phylogenetic tree based on MAGs and representative isolates' genomes from *Bartonella.* **Figure S7.** Whole-genome phylogenetic tree based on MAGs and representative isolates' genomes from *Lactobacillus* Firm4. **Figure S8.** Whole-genome phylogenetic tree based on MAGs and representative isolates' genomes from *Lactobacillus kunkeei.* **Figure S9.** Whole-genome phylogenetic tree based on MAGs and representative isolates' genomes from *Acetobacteraceae.* **Table S1.** The bacterial concentration is required to generate a given volume of droplets.

**Additional file 2: Video S1.** Generation of microfluidic droplets.

**Additional file 3: Video S2.** Morphologies of gut bacteria in droplets after cultivation.

**Additional file 4: Dataset S1.** Information of metagenome-assembled genomes.

**Additional file 5: Dataset S2.** Reports for homologous gene cluster analysis of the novel cluster from *Bifidobacterium*.

**Additional file 6: Dataset S3.** Reports for homologous gene cluster analysis of *Lactobacillus panisapium*.

## Data Availability

Sequencing data of the metagenomes and the Metagenome-assembled genomes have been deposited under BioProject PRJNA82536.

## Abbreviations

PFO: 1H,1H,2H,2H-Perfluoro-1-octanol
DAPI: Diamidino-2-phenylindole
CTAB: Cetyl trimethylammonium bromide
MIDAS: Metagenomic intra-species diversity analysis system
SNP: Single nucleotide polymorphism
SNV: Single nucleotide variant
BHI: Brain heart infusion
ANI: Whole-genome average nucleotide identity
MAG: Metagenome-assembled genome
PCoA: Principal coordinates analysis
