# Supplementary material for: Strain-level profiling with picodroplet microfluidic cultivation reveals host-specific adaption of honeybee gut symbionts
Source: Microbiome. 2022 Aug 31;10:140. doi: 10.1186/s40168-022-01333-9 (PMC9429759; doi:10.1186/s40168-022-01333-9)
Supplement: Supplementary file 2 — Additional file 1: Figure S1. Devices for microfluidic droplet generation. The microfluidic droplets of bacterial cells were generated by the droplet entrapping microfluidic cell-sorter integrating with two pressure pumps. The chip consists of two inlets for continuous and aqueous phases and one outlet for collecting the water-in-oil emulsions. The two liquids confined within microfluidic channels are brought together at the flow-focusing junction, and the generated droplets were collected in a Teflon tube. Figure S2. Poisson distribution curve. When the average number of cells per droplet (λ) is determined, the distribution of the number of bacteria in the droplet conforms to the Poisson distribution. Figure S3. The generated droplets are introduced into Teflon tubes for incubation. Figure S4. (A-B) The Shannon (A) and Simpson (B) diversity metrics of the data sets. (C-D) Microbiome compositions in different developmental stages are plotted on Jaccard (C) and Bray-Curtis (D) PCoA graphs. Figure S5. Whole-genome phylogenetic tree based on MAGs and representative isolates' genomes from Apibacter. Figure S6. Whole-genome phylogenetic tree based on MAGs and representative isolates' genomes from Bartonella. Figure S7. Whole-genome phylogenetic tree based on MAGs and representative isolates' genomes from Lactobacillus Firm4. Figure S8. Whole-genome phylogenetic tree based on MAGs and representative isolates' genomes from Lactobacillus kunkeei. Figure S9. Whole-genome phylogenetic tree based on MAGs and representative isolates' genomes from Acetobacteraceae. Table S1. The bacterial concentration is required to generate a given volume of droplets. [file 40168_2022_1333_MOESM1_ESM.pdf]

Supplementary Information

Strain-level profiling with picodroplet microfluidic cultivation reveals host-specific adaption of honeybee gut symbionts

Yujie Meng[1], Shuang Li[2], Chong Zhang[2,*], Hao Zheng[1,*]

[1]College of Food Science and Nutritional Engineering, China Agricultural University, Beijing 100083, China

[2]Department of Chemical Engineering, Institute of Biochemical Engineering, Tsinghua University, Beijing 100084, China

*Corresponding authors:

Hao Zheng, hao.zheng@cau.edu.cn

Chong Zhang, chongzhang@tsinghua.edu.cn

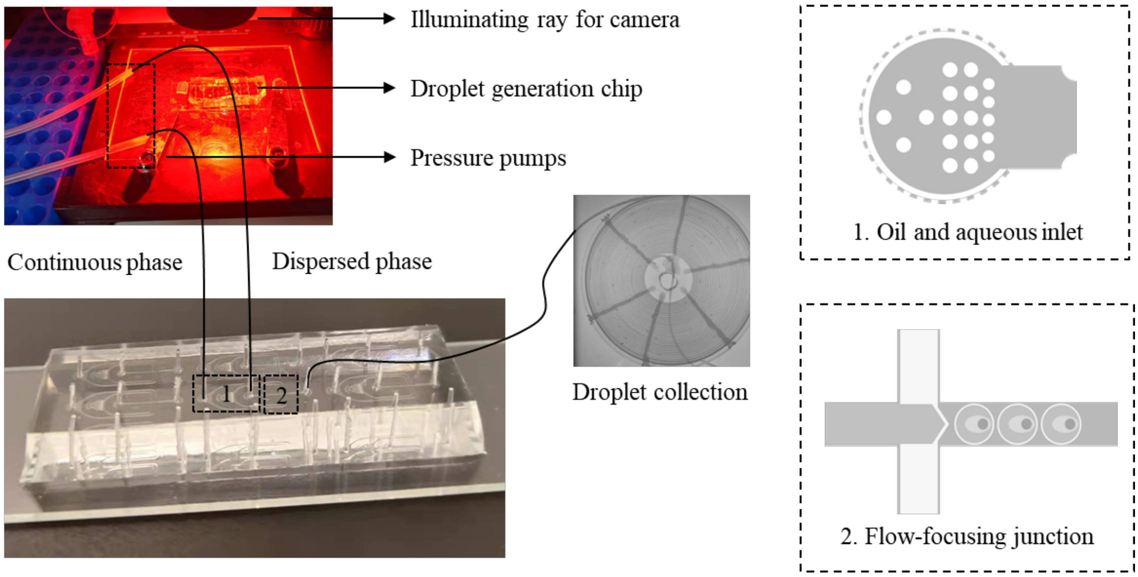

Figure S1. Devices for microfluidic droplet generation. The microfluidic droplets of bacterial cells were generated by the droplet entrapping microfluidic cell-sorter integrating with two pressure pumps. The chip consists of two inlets for continuous and aqueous phases and one outlet for collecting the water-in-oil emulsions. The two liquids confined within microfluidic channels are brought together at the flow-focusing junction, and the generated droplets were collected in a Teflon tube.

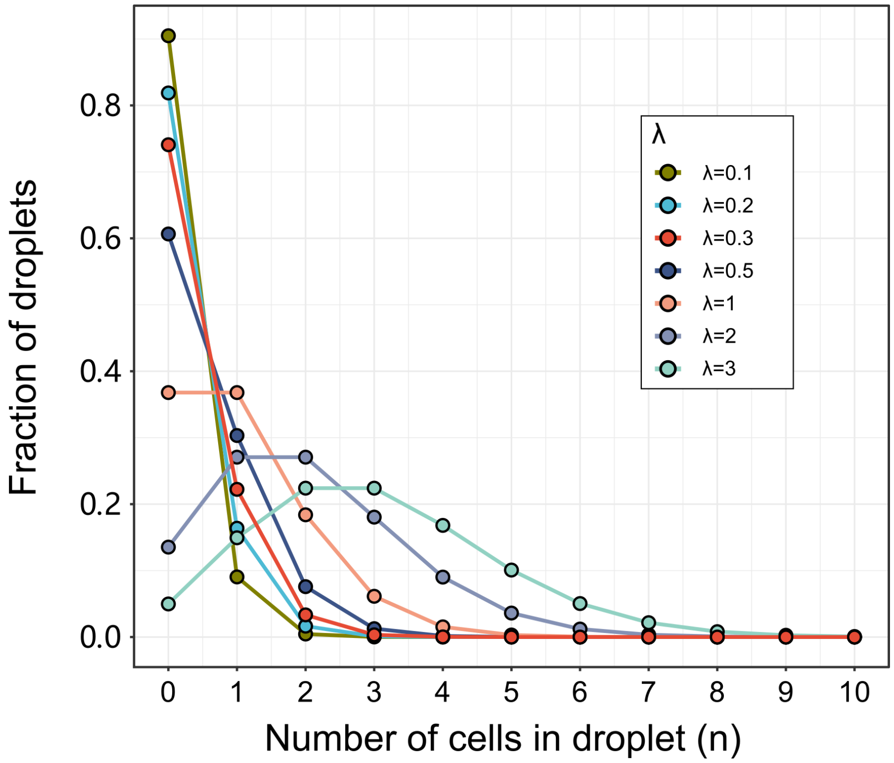

Figure S2. Poisson distribution curve. When the average number of cells per droplet (λ) is determined, the distribution of the number of bacteria in the droplet conforms to the Poisson distribution.

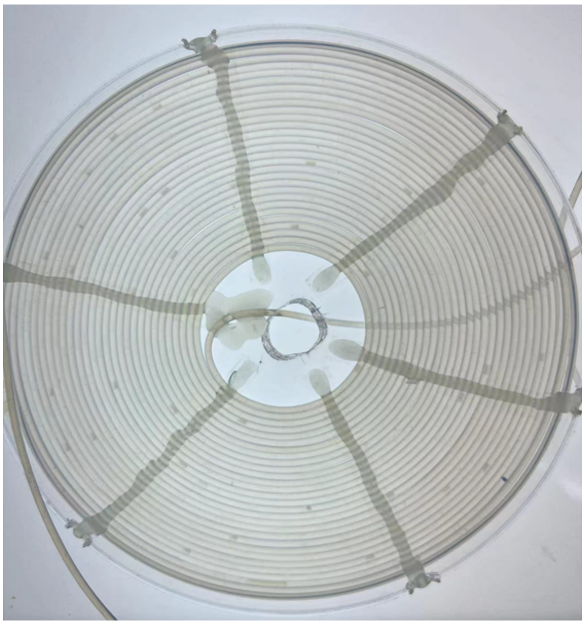

Figure S3. The generated droplets are introduced into Teflon tubes for incubation.

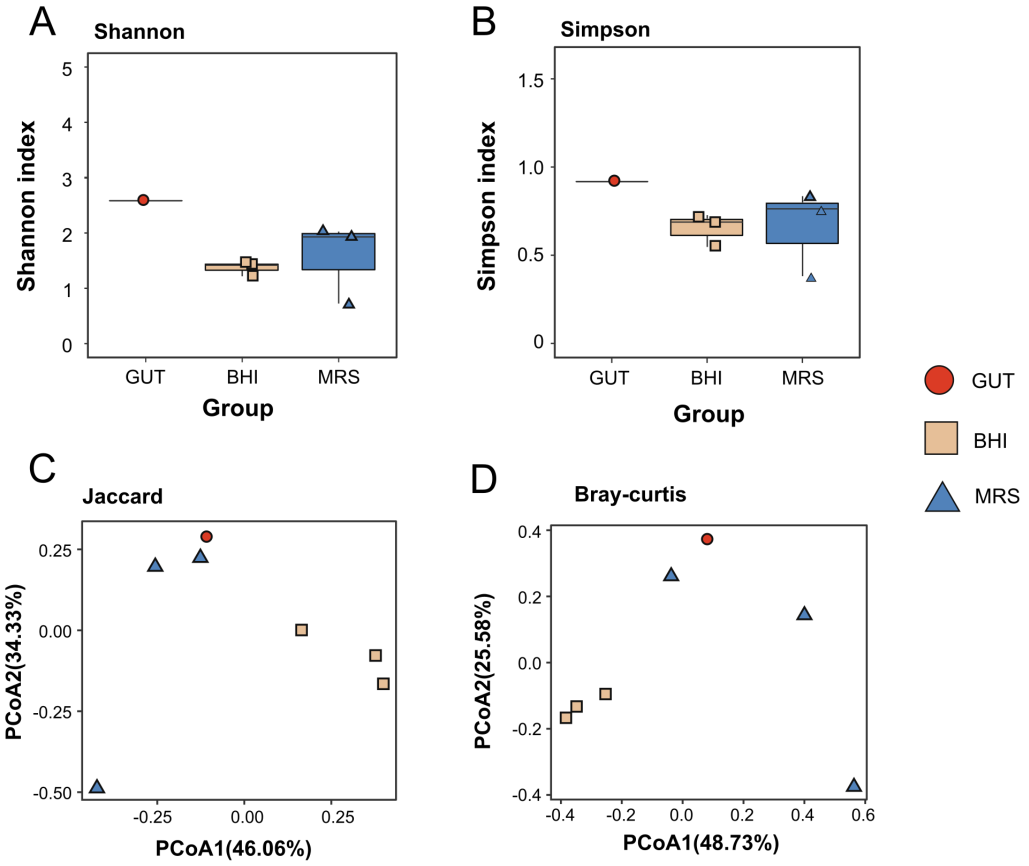

Figure S4. (A-B) The Shannon **(A)** and Simpson **(B)** diversity metrics of the data sets. **(C-D)** Microbiome compositions in different developmental stages are plotted on Jaccard **(C)** and Bray-Curtis **(D)** PCoA graphs.

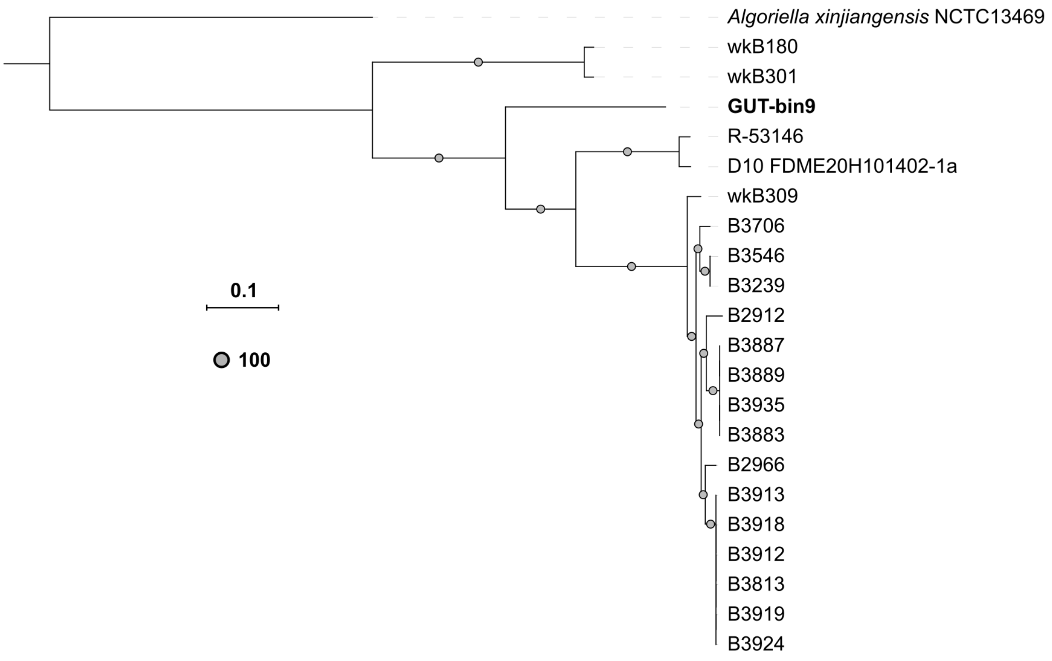

Figure S5. Whole-genome phylogenetic tree based on MAGs and representative isolates' genomes from *Apibacter*.

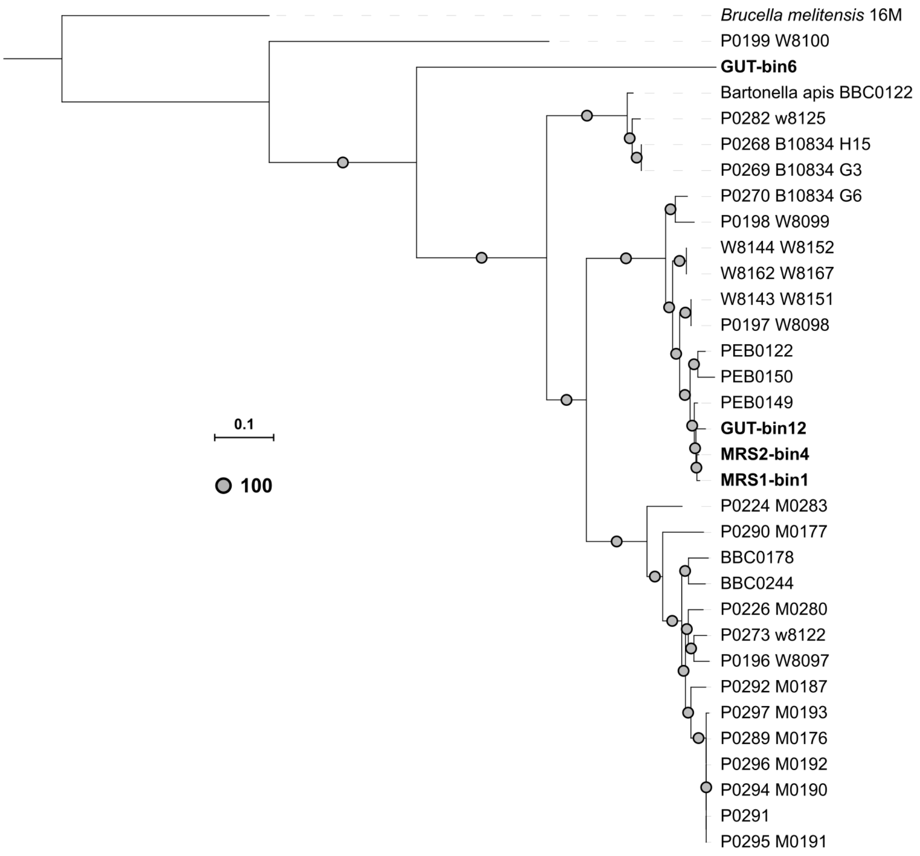

Figure S6. Whole-genome phylogenetic tree based on MAGs and representative isolates' genomes from *Bartonella*.

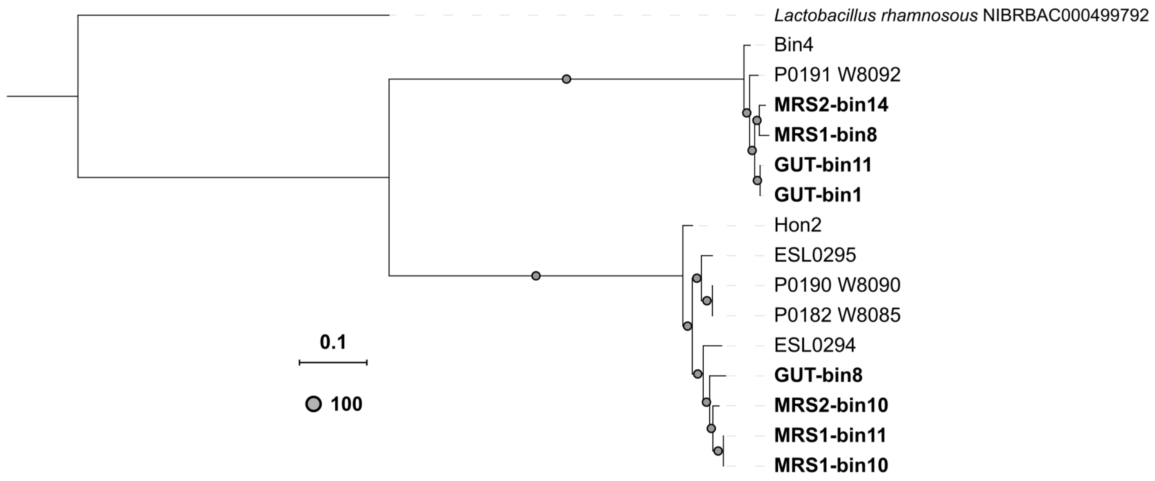

Figure S7. Whole-genome phylogenetic tree based on MAGs and representative isolates' genomes from *Lactobacillus* Firm4.

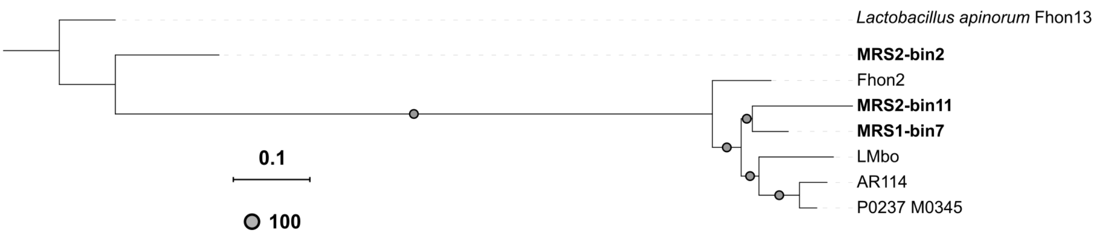

Figure S8. Whole-genome phylogenetic tree based on MAGs and representative isolates' genomes from *Lactobacillus kunkeei*.

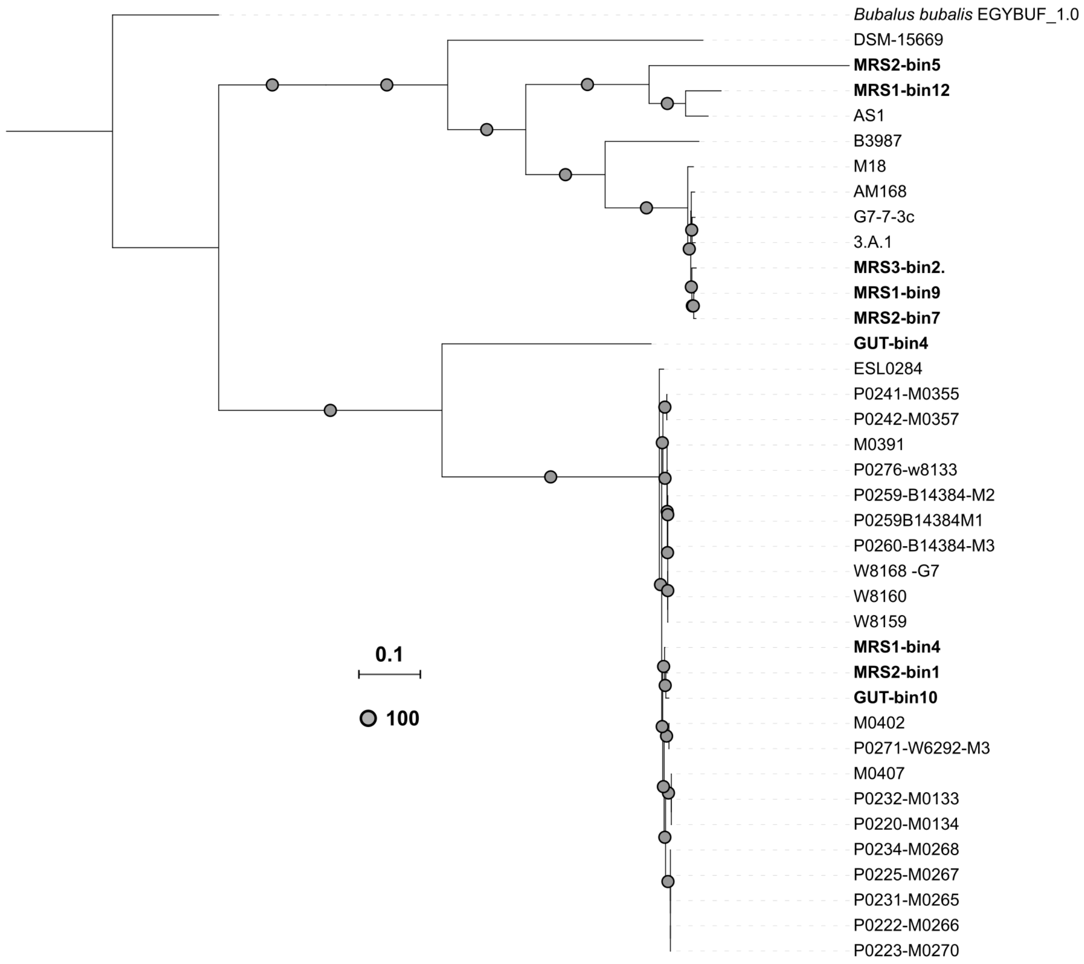

Figure S9. Whole-genome phylogenetic tree based on MAGs and representative isolates' genomes from *Acetobacteraceae*.

Table S1. The bacterial concentration is required to generate a given volume of droplets.

Diameter of droplets (μm)	The volume of droplets (pL)	Bacterial concentration (CFU/mL)
10	0.523	5.736×10^{8}
15	1.766	1.699×10^{8}
20	4.533	6.618×10^{7}
25	8.177	3.669×10^{7}
30	14.13	2.123×10^{7}
40	33.493	8.957×10^{6}

Additional files:

Additional file 1: Dataset S1. Information of metagenome-assembled genomes.

Additional file 2: Dataset S2. Reports for homologous gene cluster analysis of the novel cluster from *Bifidobacterium*.

Additional file 3: Dataset S3. Reports for homologous gene cluster analysis of *Lactobacillus panisapium*.

Additional file 4: Video S1. Generation of microfluidic droplets.

Additional file 5: Video S2. Morphologies of gut bacteria in droplets after cultivation.
